# Microencapsulation in Alginate and Chitosan Microgels to Enhance Viability of *Bifidobacterium longum* for Oral Delivery

**DOI:** 10.3389/fmicb.2016.00494

**Published:** 2016-04-19

**Authors:** Timothy W. Yeung, Elif F. Üçok, Kendra A. Tiani, David J. McClements, David A. Sela

**Affiliations:** ^1^Department of Food Science, University of MassachusettsAmherst, MA, USA; ^2^Commonwealth Honors College, University of MassachusettsAmherst, MA, USA; ^3^Center for Bioactive Delivery, Institute of Applied Life Science, University of MassachusettsAmherst, MA, USA; ^4^Center for Microbiome Research, University of Massachusetts Medical SchoolWorcester, MA, USA

**Keywords:** microencapsulation, bifidobacteria, probiotics, simulated digestion, oral delivery

## Abstract

Probiotic microorganisms are incorporated into a wide variety of foods, supplements, and pharmaceuticals to promote human health and wellness. However, maintaining bacterial cell viability during storage and gastrointestinal transit remains a challenge. Encapsulation of bifidobacteria within food-grade hydrogel particles potentially mitigates their sensitivity to environmental stresses. In this study, *Bifidobacterium longum* subspecies and strains were encapsulated in core-shell microgels consisting of an alginate core and a microgel shell. Encapsulated obligate anaerobes *Bifidobacterium longum* subsp. *infantis* and *Bifidobacterium longum* subsp. *longum* exhibited differences in viability in a strain-dependent manner, without a discernable relationship to subspecies lineage. This includes viability under aerobic storage conditions and modeled gastrointestinal tract conditions. Coating alginate microgels with chitosan did not improve viability compared to cells encapsulated in alginate microgels alone, suggesting that modifying the surface charge alone does not enhance delivery. Thus hydrogel beads have great potential for improving the stability and efficacy of bifidobacterial probiotics in various nutritional interventions.

## Introduction

Beneficial bacteria are often incorporated into functional foods and nutritional interventions to be ingested orally as probiotics. This includes humans and livestock animals that receive direct-fed microbials to enhance health outcomes and reduce pathogen load (Braat et al., 2006; Puccio et al., 2007; Neal-McKinney et al., 2012; Watson and Preedy, 2015). *Bifidobacterium longum* colonizes the human gastrointestinal tract (GIT) and is one of the 48 recognized taxa that are encompassed within the genus *Bifidobacterium* (Milani et al., 2014; Sun et al., 2015). This obligate anaerobe is one of the earliest colonizers of the infant GIT, and is present in lower concentrations in the adult gut (Schell et al., 2002; Sela et al., 2008). The bifidobacterial taxa *longum, infantis*, and *suis* were previously classified as discrete species, but more recently they were reclassified as subspecies of *B. longum* (Sakata et al., 2002). Their unification as a single species is based primarily on genomic and phenotypic similarities shared between these groups. While *Bifidobacterium animalis* subsp. *lactis* is often used for probiotic applications, *B. longum* strains are of particular interest due to its likely co-evolution with humans. This is evident in *B. longum* utilization of human milk oligosaccharides and establishes a protective gut microbiome in infants through adulthood (Sela and Mills, 2010).

*Bifidobacterium longum* is deployed in several probiotic applications using a variety of delivery formats (Adhikari et al., 2000; Fortin et al., 2011; Amine et al., 2014; Lewis et al., 2015). A relatively large dose of probiotics is recommended to impart health benefits, typically 10^6^–10^7^ CFU/g per day (Krasaekoopt et al., 2003; Roy, 2005). However, the direct incorporation of free probiotic cells into food products and supplements results in a significant decrease in cell viability throughout storage and gastrointestinal transit (Sultana et al., 2000; de Vos et al., 2010). Therefore, prolonged storage and the process of ingesting these probiotics may reduce their viability below recommended levels to achieve health benefits. Microencapsulating probiotic cells within hydrogel matrices protects them against extrinsic environmental factors thereby enhancing bacterial survival during processing, storage, and digestion (de Vos et al., 2010; Fareez et al., 2015; Yeung et al., 2016). Encapsulation may also dictate the controlled release of the probiotic at the precise anatomical site of activity within the GIT, thereby enhancing the efficacy of the probiotic through specific targeting after oral delivery (de Barros et al., 2015; Zhang et al., 2015b).

Several biopolymer materials are available to encapsulate microbes in hydrogel matrices, depending on the desired physicochemical properties of the delivery vehicle. The most commonly used food-grade biopolymers are proteins (e.g., whey proteins and caseins) and carbohydrates (e.g., starch and gums; Bagchi et al., 2010; Gaonkar et al., 2014; Etchepare et al., 2015). For many food applications, it is advantageous to encapsulate probiotics within hydrogel beads that trap bacteria within small particles containing cross-linked biopolymer molecules. These microgels must be engineered to encapsulate high concentrations of probiotics and protect them from environmental stresses, such as acidic pH, bile salts, and digestive enzymes (Zhang et al., 2015a). Alginate has been widely used as a biopolymer suitable for food applications as it is relatively inexpensive, easy to gel, biodegradable, and compatible with many food systems (Gombotz and Wee, 2012; Lee and Mooney, 2012). Indeed, recently studies have shown that lactococcal-based probiotics can be encapsulated within alginate microgels to improve their stability (Yeung et al., 2016).

There are appreciable differences between probiotic strain tolerance toward environmental and gastrointestinal stresses. Consequently, it is possible to identify particular strains that are more resistant to these stresses than others, which are therefore more suitable for commercial application (Godward et al., 2000; Krasaekoopt et al., 2004; Capela et al., 2006). As an anaerobe, bifidobacterial species including *B. longum* differ in their sensitivity to oxygen exposure and other environmental stresses during the preparative phase prior to probiotic deployment (Kawasaki et al., 2006; Ruiz et al., 2012). Therefore, bifidobacterial probiotics may be encapsulated to restrict oxidative damage during preparation and storage and to limit exposure to degradative processes within the GIT.

The aim of this study was to design, fabricate, and characterize a food-grade encapsulation system to protect *B. longum* cells during simulated storage and gastrointestinal passage. Previously, we demonstrated that encapsulation of probiotics within alginate microgels could improve their viability during storage (Yeung et al., 2016). In the current study, we encapsulated *B. longum* cells within alginate beads to determine if their viability could be enhanced in storage and gastrointestinal transit. Moreover, the impact of coating these alginate beads with a layer of chitosan was investigated as well. Chitosan coated alginate beads have previously been used to enhance the mucoadhesive properties of probiotic bacteria (Chen et al., 2013).

## Materials and Methods

### Preparation of Bacterial Cultures

Four strains of both *Bifidobacterium longum* subsp. *longum* (*B. longum*) and *Bifidobacterium longum* subsp. *infantis* (*B. infantis*) were studied (**Table 1**). All strains were originally isolated from infant feces. Stock solutions were maintained by storing bacteria at -80°C in deMann, Regosa, Sharpe (MRS) media with 0.05% L-cysteine in 25% glycerol. Bacteria were propagated in MRS with L-cysteine at 37°C for 24 h, checked for purity, and maintained on MRS agar anaerobically. Anaerobic conditions were maintained in a double chamber anaerobic hood with an airlock (88% N_2_, 10% CO_2_, and 2% H_2_) from Coy Laboratory Products (Grass Lake, Mississippi, USA).

**Table 1:** *Bifidobacterium longum* strains selected for encapsulation.

Subspecies	Strain designation
*infantis*	UMA 298
	UMA 299
	UMA 300
	UMA 305
*longum*	UMA 306
	UMA 318
	UMA 401
	UMA 402

Isolated colonies were routinely propagated in MRS broth (50 mL) for 40 h at 37° C. Cells were harvested by centrifugation at 4000 × *g* for 10 min, washed twice with 0.85% NaCl (physiological saline) solution (25 mL), and suspended in 0.85% NaCl (2 mL). The resulting cell suspensions were used either directly for assessing survival of free cells (i.e., no encapsulation) or subjected to encapsulation as described in section “Microencapsulation of Bifidobacterial Cells.” Free cell suspensions (2 mL) were stored in 0.85% NaCl solution (50 mL) at 2–5°C for up to 5 weeks to model long-term storage conditions.

### General Chemicals Used in Encapsulation and Modeled Digestion

For bacterial culture preparation, MRS broth was obtained from Becton Dickinson and Company (Sparks, MD, USA). Agar, L-cysteine hydrochloric acid, and sodium chloride (NaCl) were purchased from Sigma–Aldrich (St. Louis, MO, USA). Glycerol and sodium citrate dihydrate was purchased from Fisher Scientific (Fair Lawn, NJ, USA).

For encapsulation experiments, sodium alginate (TICA-algin HG 400 powder) was donated by TIC Gums (White Marsh, ML, USA). Calcium chloride hexahydrate, chitosan (medium molecular weight) was obtained from Sigma-Aldrich. Glacial acetic acid was purchased from Fisher Scientific.

For simulated digestion, ammonium nitrate, bile extract porcine, lipase from porcine pancreas type II, pepsin from porcine gastric mucosa, porcine gastric mucin type II, potassium chloride, potassium citrate, potassium phosphate, sodium DL-lactate, sodium hydroxide (NaOH), and uric acid sodium salt were also purchased from Sigma-Aldrich. Hydrochloric acid (HCl), phosphate buffer saline (PBS), and urea were purchased from Fisher Scientific.

### Microencapsulation of Bifidobacterial Cells

Bifidobacteria were encapsulated within alginate microgels using an injection–gelation method (Whelehan and Marison, 2011; Seiffert, 2013). Briefly, 1% (w/v) sodium alginate solution was prepared, autoclaved, and then cooled to ambient temperature. The sterile alginate solution (198 mL) was mixed with 2 mL of ∼10^9^ CFU/mL probiotic organisms suspended in physiological saline. The polymeric solution was agitated to uniformly distribute cells throughout the mixture. The alginate beads were prepared aseptically using an encapsulator (Büchi B-390^®^, Büchi Labortechnik AG, Flawil, Switzerland) with a nozzle size of 120 μm, using the manufacturer’s standard operating conditions (amplitude 3, frequency 800 Hz, electrode 800 V, pressure 250–300 mbar). Aliquots of probiotic/alginate solution were injected into 0.1 M calcium chloride solution (350 mL). After 1-h gelation under agitation, the resulting calcium alginate beads were collected by filtration, rinsed with sterile deionized water (200 mL), and re-filtered. Microbeads (∼30 mL) were stored in physiological saline solution (50 mL) at 4°C for up to 4 weeks to model long-term storage conditions. This process was repeated for all eight strains of bifidobacteria.

Unfilled alginate beads were prepared identically but without the addition of bacterial strains to the alginate solution. 1% alginate solution (200 mL) was extruded into of 0.1 M CaCl_2_ (350 mL) solution under continuous agitation. The working parameters (nozzle diameter, frequency, charge, and pressure), filtering steps and storage conditions used were the same as those for the preparation of filled alginate beads.

An aqueous chitosan solution (0.4% w/v) was prepared as described previously by Zhou et al. (1998). Briefly, chitosan (0.4 g) was dissolved in distilled water (90 mL) and glacial acetic acid (0.8 mL). The pH was adjusted to 5.0–5.1 with NaOH, and the total volume was adjusted to 100 mL. The solution was autoclaved and filtered to remove undissolved solids. Subsequently, the alginate beads were submerged in the chitosan solution to provide a secondary coating by electrostatic attraction of the cationic chitosan molecules to the surfaces of the anionic alginate beads. The mixture was agitated for 1 h before filtering and rinsing beads with sterile distilled water. Chitosan-coated alginate beads were then stored and analyzed as described in section “Alginate and Chitosan-coated Alginate Microbead Characterization.”

### Alginate and Chitosan-Coated Alginate Microbead Characterization

#### Particle Size Distribution

The particle size distribution was determined by static light scattering (Mastersizer S, Malvern Instruments, Worcestershire, UK). Each sample (1–2 mL) was suspended in distilled water (10 mL) and vortexed to avoid multiple scattering effects and to ensure homogeneity prior to analysis. Volume-weighted (D [4,3]) and surface-weighted (D [3,2]) mean particle diameters were obtained for all samples.

#### Optical Microscopy Characterization

The overall appearance of alginate and chitosan-coated alginate beads was characterized with an optical microscope (C1 Digital Eclipse, Nikon, Tokyo, Japan). Microgel suspensions (1–2 mL) were immersed in physiological saline (10 mL) and vortexed to separate individual beads. Optical images were obtained using a digital camera and further analyzed using the instrument software (EZ CSI version 3.8, Nikon).

#### Scanning Electronic Microscopy (SEM)

The bead microstructure was characterized using a bench-top scanning electron microscope (JCM-6000 NeoScope, JEOL, Tokyo, Japan). To prepare the samples prior to analysis, alginate, and chitosan-coated alginate beads were freeze-dried and sputter-coated with gold (10 nm) before loading onto the microscope. Images of the microgels were documented in representative fields.

#### Electrical Properties

The surface potential (ζ-potential) of alginate and chitosan-coated alginate microgels was evaluated by electrophoretic light scattering (Zetasizer Nano ZS, Malvern Instruments, Worcestershire, UK). For each sample, refrigerated microgels (1–2 mL) were suspended in distilled water (10 mL) and vortexed to separate the beads. Samples were then loaded into the measurement cells and analyzed.

### Modeled Long-term Storage Conditions of Encapsulated Bifidobacteria

Total cell counts of free and encapsulated bifidobacteria were determined by a modified drop plate method as previously described (Herigstad et al., 2001). Briefly, 10 drops (10 μL) of a dilution within a series (10^0^–10^7^) were deposited on MRS agar plates and counted after incubation under anaerobic conditions at 37°C.

To determine viable counts of the encapsulated bacteria, beads (1 mL) were re-suspended in 10% sodium citrate dihydrate solution (9 mL) followed by vortexing. The number of released cells was determined by plate count using MRS agar, dilutions of dissolved beads (10^-1^–10^-7^) were plated in duplicate and incubated at 37°C anaerobically for 40 h. For lower viability samples later, beads (2 mL) were re-suspended in 10% sodium citrate dihydrate solution (2 mL) instead, and dilutions (10^0^–10^-3^) were plated as before. Samples were taken over a 4-week period on days 0 (initial), 1, 3, 5, 7, 10, 14, 21, and 28. Day 24 was also plated for free cell samples.

### *In Vitro* Simulated Digestion of Alginate and Chitosan-Coated Alginate Microbeads

Fluids used in *in vitro* modeling of digestion were prepared based on the method described by Li et al. (2011). One liter of modeled saliva stock solution was prepared with ammonium nitrate (0.328 g), potassium chloride (0.202 g), potassium citrate (0.308 g), potassium phosphate (0.636 g), sodium chloride (1.594 g), sodium DL-lactate (0.146 g), urea (0.198 g), and uric acid sodium salt (0.021 g) in distilled water. The stock solution was then filter-sterilized. The day before digestion experiments were carried out, the salivary phase was prepared by adding porcine gastric mucin type II (2.4 g) to saliva stock solution (80 mL). The solution was stirred overnight at room temperature to completely dissolve the powder.

One liter of simulated gastric stock solution was prepared by adding sodium chloride (2 g) and 6 M hydrochloric acid (7 mL) to distilled water and filter sterilizing. The simulated intestinal stock solution (500 ml) was prepared by adding calcium chloride hexahydrate (27.38 g) and sodium chloride (109.685 g) to distilled water and autoclaved. Pepsin extracted from porcine gastric mucosa (0.32 g) was then added to gastric stock solution (100 mL).

The day before digestion experiments were carried out, porcine bile extract (0.75 g) was added to PBS solution (14 mL) for the modeled intestinal phase. The solution was stirred overnight at room temperature to completely dissolve the powder. Lipase from porcine pancreas type II (0.24 g) was dissolved in PBS solution (10 mL); the solution (5 mL) was then added with bile salt solution (7 mL) and intestinal stock solution (33 mL).

Free and encapsulated bifidobacteria cells were separately added to simulated saliva fluids (22 mL, pH adjusted to 6.7–6.8), simulated gastric fluids (45 mL, pH adjusted to 2.5–2.6) or simulated intestinal fluids (45 mL, pH adjusted to 7.0–7.2) and stored in a shaking incubator (MaxQ 6000, Thermo Scientific, Waltham, MA, USA) set at 37°C with a shaking speed of 110 rpm (Supplementary Figure S1). Dilutions (10^0^–10^5^) were plated on MRS agar for initial, 5, 10, 15, or 30 min exposure and incubated anaerobically for at least 48 h.

### Statistical Analysis

The mean of two or three individual determinations was used to calculate particle size, ζ-potential. The mean of 10 replicate drops was used to calculate cell counts. Analysis of variance (ANOVA) followed by Tukey honest significant difference test was use to analyze all data and compare individual means. This was performed using statistical software (GraphPad Prism 6, GraphPad Software, La Jolla, CA, USA).

## Results

### Particle Size Analysis of Alginate and Chitosan-Coated Alginate Microgels

Light scattering was used to determine the mean particle diameter of the different microgel samples (**Table 2**). The mean particle sizes of alginate beads containing similar strains were similar, ranging from 135 to 185 μm (D [3,2]) for encapsulated *B. infantis* strains and 149–216 μm (D [3,2]) for encapsulated *B. longum* strains. The chitosan-coated alginate beads were significantly larger compared than the alginate beads, ranging from 191 to 292 μm (D [3,2]). This increase in particle size may have been because of the additional coating formed by the alginate molecules, or because of some aggregation of the microgels. Microgel aggregation may have occurred due to bridging flocculation, which is the ability of the chitosan cation to adsorb to the surfaces of two or more anionic alginate beads. Additional information regarding the structural configuration of the microgels was therefore obtained through microscopy.

**Table 2:** Volume-based (D [4,3]) and surface-based (D [3,2]) mean particle diameters measured by static light scattering alginate and chitosan-coated alginate beads with strains of bifidobacteria.

Beads		μm	
		D [4,3]	D [3,2]
**Alg.**			
Subsp. *infantis*	UMA 298	233 ± 4^ab^	167 ± 6^abc^
	UMA 299	230 ± 3^ab^	162 ± 3^b^
	UMA 300	251 ± 6^a^	185 ± 12 ^cd^
	UMA 305	211 ± 4^b^	135 ± 2^e^
Subsp. *longum*	UMA 306	247 ± 13^ac^	164 ± 4^ab^
	UMA 318	228 ± 11^ab^	149 ± 13^abe^
	UMA 401	277 ± 4^cd^	216 ± 3^f^
	UMA 402	287 ± 13^de^	195 ± 3^dfg^
**Chit.-alg.**			
Subsp. *infantis*	UMA 299	327 ± 2^fg^	292 ± 3^h^
	UMA 300	344 ± 14^f^	237 ± 3^i^
Subsp. *longum*	UMA 401	310 ± 20^eg^	213 ± 4^f^
	UMA 402	315 ± 26^efg^	191 ± 11^g^

*Values are shown as average particle size ± standard deviation. Values followed by the same letters in the same column are not significantly different (p > 0.05) from each other.*

### Optical Microscopy of Alginate and Chitosan-Coated Alginate Microbeads

**FIGURE 1:**
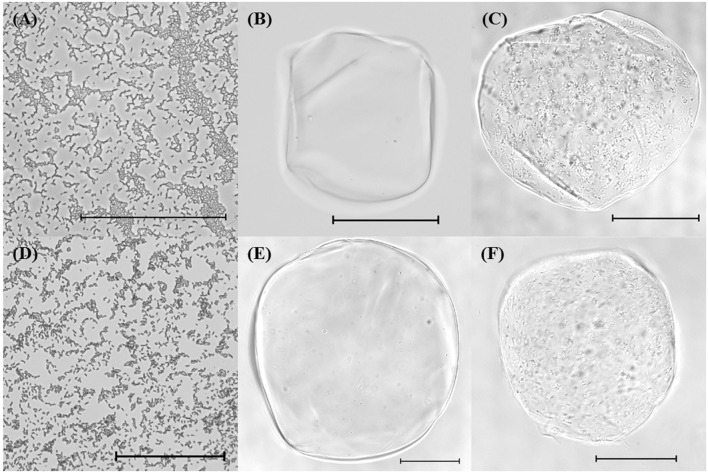
**Optical microscope images of (A) *Bifidobacterium longum* subsp. *infantis* UMA 300 (20×), (B) unfilled alginate bead (20×), (C) filled alginate bead with *B. longum* subsp. *longum* UMA 306 (20×), (D) *B. longum* subsp. *longum* UMA 318 (20×), (E) unfilled chitosan-coated alginate bead (20×), (F) filled chitosan-coated alginate bead with *B. longum* subsp. *infantis* UMA 299 (20×).** Scale bars represent 100 μm.

The structures of samples containing free cells or bacterial-loaded microgels were determined using optical microscopy immediately after encapsulation (**Figure 1**). Free cells appeared rod-shaped as expected for bifidobacteria (**Figures 1A,D**). The unfilled alginate and chitosan-coated alginate microgels were similar in morphology, although the individual coated alginate beads did appear larger than the uncoated ones, which is consistent with the particle size analysis (**Figures 1C,F**). Encapsulated bifidobacteria were clearly visualized within the microgels for both alginate and chitosan-coated alginate microgels (**Figures 1E,F**). The bifidobacterial-loaded alginate and chitosan-coated alginate beads had a similar external appearance as the equivalent unloaded beads. The microgels were generally spherical with diameters around 100–300 μm for all samples.

### Scanning Electron Microscopy

**FIGURE 2:**
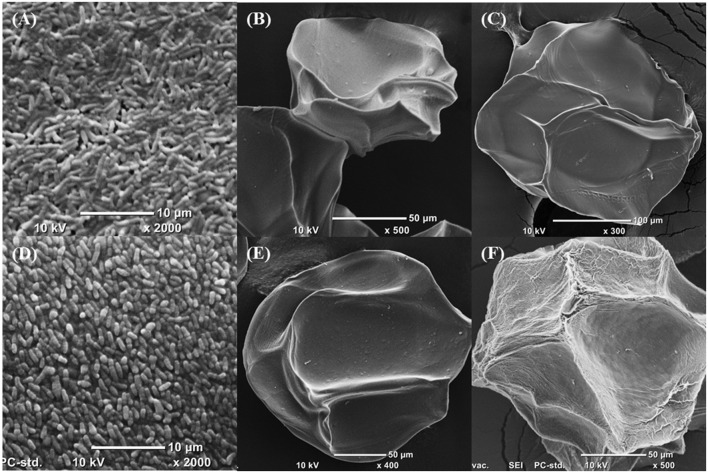
**Scanning electron micrographs of (A) *B. longum* subsp. *infantis* UMA299, (B) unfilled alginate bead, (C) unfilled chitosan-coated alginate bead, (D) *B. longum* subsp. *longum* UMA 306, (E) alginate bead containing *B. longum* subsp. *longum* UMA 401, (F) chitosan-coated alginate bead containing *B. longum* subsp. *longum* UMA 300.** Samples were dried before sputter-coating with gold. SEM was set at high-vacuum, 10 kV.

Scanning electron microscopy (SEM) was used to inspect the structure of the alginate and chitosan-coated alginate beads (**Figure 2**). Freeze-dried microgels were uniform in size and shape. However, the surfaces of the microgels observed by SEM appeared wrinkled, whereas they presented as smooth when observed by optical microscopy. This is likely due to sublimation of water originally trapped within the hydrogel matrix, as has been described previously (Yeung et al., 2016). The chitosan-coated alginate beads appeared to be more irregular in shape compared to alginate beads. Qualitatively, the alginate beads had smoother wrinkles and microstructures, whereas the chitosan-coated beads exhibited sharp jagged edges. This observation suggests that the chitosan layer has been successfully deposited onto the external surfaces of the alginate microgels.

### Zeta Potential Analysis of Microencapsulated Bifidobacteria

Electrophoretic light scattering was used to evaluate the electrical characteristics of the microgels (**Table 3**). The ζ-potentials of all the alginate beads were negative, ranging from -4.2 to -9.4 mV for *B. infantis* and -2.6 to -4.4 mV for *B. longum* as predicted with this coating. In contrast, all chitosan-coated alginate bead samples had positive surface potentials ranging from +9.9 to +14.9 mV for *B. infantis* and +0.8 to +9.0 mV for *B. longum*. These results indicate that the cationic chitosan molecules formed a secondary shell around the anionic calcium alginate beads.

**Table 3:** Zeta potential of alginate and chitosan-coated alginate beads with strains of bifidobacteria.

Beads		mV
**Alg.**		
Subsp. *infantis*	UMA 298	-5.23 ± 2.06^ab^
	UMA 299	-9.42 ± 2.54^a^
	UMA 300	-8.73 ± 4.88^a^
	UMA 305	-4.15 ± 1.17^ab^
Subsp. *longum*	UMA 306	-3.14 ± 2.24^ab^
	UMA 318	-2.60 ± 0.04^ab^
	UMA 401	-4.38 ± 0.64^ab^
	UMA 402	-4.28 ± 1.12^ab^
**Chit.-alg.**		
Subsp. *infantis*	UMA 299	9.92 ± 3.92^c^
	UMA 300	14.87 ± 4.26^c^
Subsp. *longum*	UMA 401	0.79 ± 2.53^bd^
	UMA 402	9.03 ± 4.90^cd^

*Values are shown as average ζ-potential ± standard deviation. Values followed by the same letters are not significantly different (p > 0.05) from each other.*

### Survival of Bifidobacterial Strains during Long-term Storage

#### Non-encapsulated Bifidobacterial Cells

**FIGURE 3:**
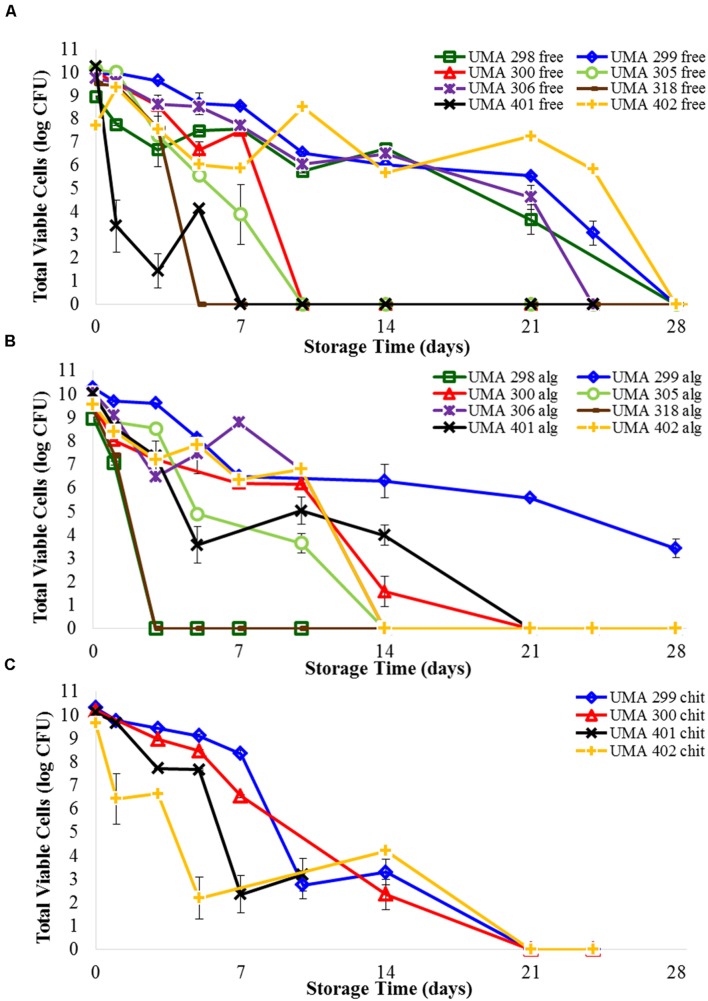
**Survival of (A) free *B. longum* cells, (B) *B. longum* cells in calcium alginate microbeads, (C) *B. longum* cells in chitosan-coated alginate microbeads in wet refrigerated storage over time.** Counts based on samples drop-plated on MRS agar and incubated at 37°C anaerobically. Error bars indicate the standard error of replicate counts (*n* = 10).

The viability of four *B. longum* and four *B. infantis* strains that were not encapsulated was determined during 5 weeks of storage (**Figure 3A**; Supplementary Table S1). As expected, there was a decrease in the viability of the bifidobacteria evaluated, but the rate of the decrease was strain dependent. A sharp decrease in viability was observed for *B. infantis* UMA318 and *B. longum* UMA401, diminishing by 9–10 log CFU over the course of a week under aerobic conditions. *B. infantis* UMA 300 and *B. infantis* UMA 305 remained viable for slightly longer, with a 10-log reduction observed within 10 days. Whereas, *B. infantis* UMA 298 and *B. infantis* 306 exhibited a 9–10 log decrease over 2 weeks of storage. Interestingly, *B. infantis* UMA 299 and *B. longum* UMA 402 survived the longest, as viable cell counts diminished by 7–8 logs over 3 weeks before decreasing to undetectable levels.

#### Encapsulated Bifidobacterial Cells

Viability following encapsulation was determined for all eight bifidobacterial strains (**Figure 3B**; Supplementary Table S2). There were distinct differences between the effects of encapsulation depending on strain type. The cell viability of *B. infantis* UMA 298, *B. infantis* UMA 305, and *B. longum* UMA 318 stains rapidly decreased and were undetectable after 3 days. Unexpectedly, *B. infantis* UMA 298 and *B. infantis* UMA 305 in alginate were inactivated faster than the corresponding free cells, being undetectable after 24 and 10 days, respectively. Viability of the encapsulated *B. longum* UMA 318 was identical to that of free cells. *B. infantis* UMA 300, *B. longum* UMA 306, *B. longum* 401, and *B. longum* 402 maintained viable populations that decreased by 3–4 log CFU after 10 days of storage before rapidly decreasing to zero. Encapsulated *B. infantis* UMA 300 survived 3 days longer than corresponding free cells; encapsulated *B. longum* UMA 401 lasted a week longer than free cells. Encapsulated *B. longum* UMA 306 survived similarly to free cells, and encapsulated *B. longum* UMA 402 survived over a week shorter than corresponding free cells. Interestingly, *B. infantis* UMA 299 viability was enhanced as it experienced a 5 log CFU reduction in 3 weeks compared to an 8 log CFU reduction during this time for the non-encapsulated cells. Thus, encapsulating with alginate extended cell viability of *B. infantis* UMA 299 and 300 by a few days, and extended viability of *B. longum* UMA 401 cells by a week. Encapsulating other *B. infantis* and *B. longum* strains did not appear to extend viability over the storage conditions used.

Two strains each of *B. infantis* (UMA 299 and 300) and *B. longum* (UMA 401 and 402) were encapsulated in a secondary coating of chitosan applied to the alginate bead core and submitted to testing over time (**Figure 3C**; Supplementary Table S3). *B. longum* UMA 401 and 402 both decreased 3–4 log within 3 days, and fell to undetectable levels by 2 weeks. Viability of *B. infantis* UMA 299 and *B. infantis* UMA 300 decreased only 2 logs in 5 days, before falling to undetectable levels after 2 weeks. Encapsulating *B. infantis* UMA 299, *B. infantis* UMA 300, and *B. longum* UMA 401 cells in chitosan-coated alginate beads did not appear to extend viability compared with uncoated alginate microbeads. *B. longum* UMA 402 cells in chitosan-coated alginate decreased 2.4 log CFU, whereas *B. longum* UMA 402 cells in alginate alone decreased 7.2 log CFU, between day 3 and day 14. Hence, encapsulating *B. longum* UMA 402 in chitosan-coated alginate extended detectible viability 4 days more than encapsulating *B. longum* UMA 402 in alginate alone.

### Survival of Encapsulated Cells during Simulated Digestion

Free and bifidobacterial cells encapsulated in chitosan-coated alginate beads were subjected to simulated digestion in a GIT model as previously described (Li et al., 2011). Free *B. infantis* UMA 299 and cells encapsulated chitosan-coated alginate were immersed separately in simulated salivary, gastric, and intestinal phases and assessed over time for cell viability (**Table 4**). The strain was selected based on its high viability during storage in free and encapsulated forms (**Table 3**; Supplementary Tables S1–S3). The bacteria appeared to be relatively stable within simulated saliva fluids, as less than a 1 log CFU reduction was experienced in 30 min of exposure regardless of encapsulation. The model salivary juice did not greatly inhibit cell viability in general, as less than one log CFU reduction was experienced in 30 min of exposure regardless of encapsulation. However, microencapsulation provided enhanced protection for UMA299 by shielding the strain from the low pH of the gastric phase. Encapsulated cells decreased by 1.4 logs CFU, whereas untreated cells decreased by 2.7 logs following exposure to pH 2.5 conditions (5 min). This indicates a significant, albeit fleeting protection afforded to the encapsulated cells as viability was abrogated after 10 min of exposure to the gastric phase. Similarly, UMA299 cell viability was not detectible after 5 min of exposure to the intestinal phase. *B. longum* UMA 402 encapsulated in chitosan-coated alginate was also subjected to simulated digestion in preliminary tests (data not shown). As with *B. infantis*, cell viability remained stable in the modeled salivary phase, but underwent a 6-log reduction after only a few minutes exposure to gastric phase (pH 2.5).

**Table 4:** Simulated digestion of free and encapsulated *B. longum* subsp. *infantis* UMA 299 in three separate stages.

	log CFU					
Time (minutes)	Free			Encapsulated		
	Saliva	Gastric	Intestinal	Saliva	Gastric	Intestinal
pH	6.74	2.53	7.04	6.78	2.57	7.12
0	9.63 ± 0.07^aA^	9.63 ± 0.07^aA^	9.63 ± 0.07^aA^	8.40 ± 0.84^abB^	8.40 ± 0.84^aB^	8.40 ± 0.84^aB^
5	9.17 ± 0.06^a^	6.99 ± 0.03^b^	ND	8.10 ± 0.05^a^	6.90 ± 0.04^a^	ND
10	9.28 ± 0.09^a^	ND	ND	7.89 ± 0.07^a^	ND	ND
15	9.14 ± 0.06^a^	ND	ND	6.42 ± 1.07^b^	ND	ND
30	9.01 ± 0.06^a^	ND	ND	8.14 ± 0.040^a^	ND	ND

*Counts based on samples drop-plated on MRS agar and incubated at 37°C anaerobically. Values are shown as mean cell number ± standard error of replicate counts (n = 10). Means within each column followed by the same lowercase letters are not significantly different (p > 0.05) from each other. Means within each row followed by the same uppercase letters are not significantly different (p > 0.05) from each other.*

## Discussion

Initially, chitosan-coating of alginate beads was postulated to enhance the viability of encapsulated probiotics by reducing their exposure to environmental stresses during storage and within the GIT (Kamalian et al., 2014). Accordingly, the influence of encapsulation on a panel of *B. longum* strains to assess differential viability was systematically studied. The calcium alginate beads formed using an injection–gelation method were roughly spherical in shape, negatively charged, and had dimensions around 130–220 μm. Coating the alginate beads with chitosan caused a small increase in their size and changed their charge from negative to positive. Optical microscopy (**Figures 1C,F**) confirmed that the bifidobacteria were immobilized within the hydrogel beads, which is consistent with previous encapsulation studies (Hansen et al., 2002; Fareez et al., 2015; Yeung et al., 2016).

Interestingly, encapsulation of bifidobacteria in chitosan-coated alginate beads led to decreased improvement in their storage or gastrointestinal stability compared with cells in alginate beads. One possible explanation for this observation is that the alginate hydrogel used had relatively large pores, and so small molecules, such as oxygen, acids, bile salts, or digestive enzymes, could easily diffuse into the microgels and inactivate the encapsulated bacteria (McClements, 2015). These results suggest that a simple secondary layer of chitosan alone will not fully protect encapsulated bifidobacteria, and that further optimization is required to engineer more effective delivery systems. Previous studies have shown that alginate has a prebiotic effect on bifidobacteria, which might account for its ability to enhanced viability, potentially through a non-encapsulation mechanism (Wang et al., 2006; Ramnani et al., 2012). In future studies, it may be useful to examine the influence of different biopolymer materials and methods on the ability of microgels to enhance probiotic viability. As an example, the hydrogel pore size may be decreased to limit molecular diffusion, with the addition of anti-oxidants to limit oxidation reactions and prebiotics to stimulate probiotic growth in the colon. Since bifidobacteria ferment oligosaccharides within the gut, a synbiotic approach that integrates prebiotic substrates including plant or milk oligosaccharides may advance bifidobacterial-based delivery (Sela, 2011). Alternatively, judicious selection of strain selection that are resistant to acids, bile salts, or digestive enzymes may enhance the delivery scheme. However, previous studies indicate that most bifidobacteria strains typically exhibit a significant decrease in survival around pH 4 which would necessitate shielding from gastric conditions (Sun and Griffiths, 2000).

Bifidobacteria have been exposed to simulated digestive fluids in previously conducted studies (O’Riordan et al., 2001; Hansen et al., 2002; Kamalian et al., 2014). Although specific strains tested and experimental schemes vary between studies. Hansen et al. (2002) encapsulated several bifidobacterial strains in microgels formed by an emulsion-templating method, and then exposed them to simulated gastric and small intestinal fluids. In this study, *B. infantis* and *B. longum* strains showed a 4–6 log CFU/mL decrease between exposure to gastric fluids set at pH 6.0 and pH 2.0 for 2 h, and 3–5 log CFU/mL reduction between exposure to intestinal fluid containing 0 and 1% bile for 24 h. Hansen et al. (2002) also encapsulated *B. longum* experienced a 5-log CFU/mL reduction after 30 min exposure to gastric juice (pH 2.0). In the study herein, *B. infantis* UMA 299 encapsulated in chitosan-coated alginate underwent an 8-log reduction in a 10-min exposure to gastric fluid (pH 2.6), and an 8-log reduction in 5 min exposure to intestinal fluid (**Figure 3C**; Supplementary Table S3). This study included 0.75% bile extract, pepsin, and lipases in the gastric and intestinal fluids for the purpose of simulating the harsh conditions of the human GIT. The bifidobacterial general stress response has been studied (Sánchez et al., 2007; Zomer et al., 2009). As with the phylogenetically dissimilar lactic acid bacteria, bifidobacteria employ ATPases to pump protons from the cell when exposed to acidic conditions (Matsumoto et al., 2004; Ventura et al., 2004). In addition, when exposed to bile during gastrointestinal transit, certain bifidobacterial strains deploy bile salt hydrolase to promote cell survival in the small intestine (Ruiz et al., 2014).

In an additional study, an emulsion encapsulation method was performed on *B. pseudocatenulatum* G4 in chitosan-coated alginate and exposed to gastric conditions (pH 1.5) for 2 h followed by intestinal phase for 5 h (Kamalian et al., 2014). The encapsulated *B. pseudocatenulatum* experienced a 4-log reduction when encapsulated in alginate and a 2-log reduction in chitosan-coated alginate, relative to the 5-log reduction in the control. However, this was accomplished in the absence of digestive enzymes or bile salts in simulated gastric and intestinal fluids that would present additional hurdles to the bifidobacterial cells. O’Riordan et al. (2001) studied spray-dried *Bifidobacterium* spp. PL1 in starch and subjected the resultant granules to simulated digestion. After 3 h of exposure to buffer with pH 2.8, they were unable to detect viable cells as well as other sampling points in between 0 and 3 h. This is consistent with the results presented in this study.

In summary, bifidobacterial viability following encapsulation varied between subspecies as well as strains. This suggests that there is a range of genotypic and phenotypic factors contributing to stress responses that promote enhanced viability. Further functional genomic analysis of encapsulated probiotic organisms can aid in matching strains with the particular encapsulation process to optimize cell integrity during storage. Moreover, similar approaches may be used in selecting ideal delivery vehicles to shield bifidobacteria during GIT transit to arrive intact and metabolically poised to exert beneficial activities in the distal colon. Subsequent formulations may optimize delivery vehicles by incorporating antioxidants and cryoprotectants within the encapsulation gel matrix to preserve bifidobacterial cell viability.

## Author Contributions

TY, DM, and DS conceived the experimental plan. TY conducted laboratory experiments, data analysis, and drafted the manuscript. EÜ and KT assisted with experiments, analyses, and contributed to the manuscript. DS supervised execution of the experimental plan, analyzed data, and critically reviewed the final manuscript. All authors read and approved the manuscript prior to submission.

## Conflict of Interest Statement

The authors declare that the research was conducted in the absence of any commercial or financial relationships that could be construed as a potential conflict of interest.
