# Supplementary material for: Microencapsulation in Alginate and Chitosan Microgels to Enhance Viability of Bifidobacterium longum for Oral Delivery
Source: Front Microbiol. 2016 Apr 19;7:494. doi: 10.3389/fmicb.2016.00494 (PMC4835488; doi:10.3389/fmicb.2016.00494)
Supplement: Supplementary file 1 [file Data_Sheet_1.PDF]

Supplementary Material

Microencapsulation in alginate and chitosan microgels to enhance viability of *Bifidobacterium longum* for oral delivery

Timothy W. Yeung[1], Elif F. Üçok[1], Kendra A. Tiani[1, 2], D. Julian McClements[1, 3], and David A. Sela[1, 3, 4*]

[1] Department of Food Science, University of Massachusetts, Amherst, MA, USA
[2] Commonwealth Honors College, University of Massachusetts, Amherst, MA, USA
[3] Center for Bioactive Delivery, Institute of Applied Life Science, University of Massachusetts, Amherst, MA, USA
[4] Center for Microbiome Research, University of Massachusetts Medical School, Worcester, MA, USA

***Correspondence:** David A. Sela, davidsela@umass.edu

Figure S1. Experimental design schematic for simulated digestion of free and encapsulated *B. infantis* UMA 299. Samples were exposed to salivary (containing mucin, pH 6.7-6.8), gastric (containing hydrochloric acid and pepsin, pH 2.5-2.6), and intestinal (containing bile salts and lipase, pH 7.0-7.2) phases separately. One milliliter samples were obtained at times 0 (before exposure), 5, 10, 15, and 30 minutes exposure.

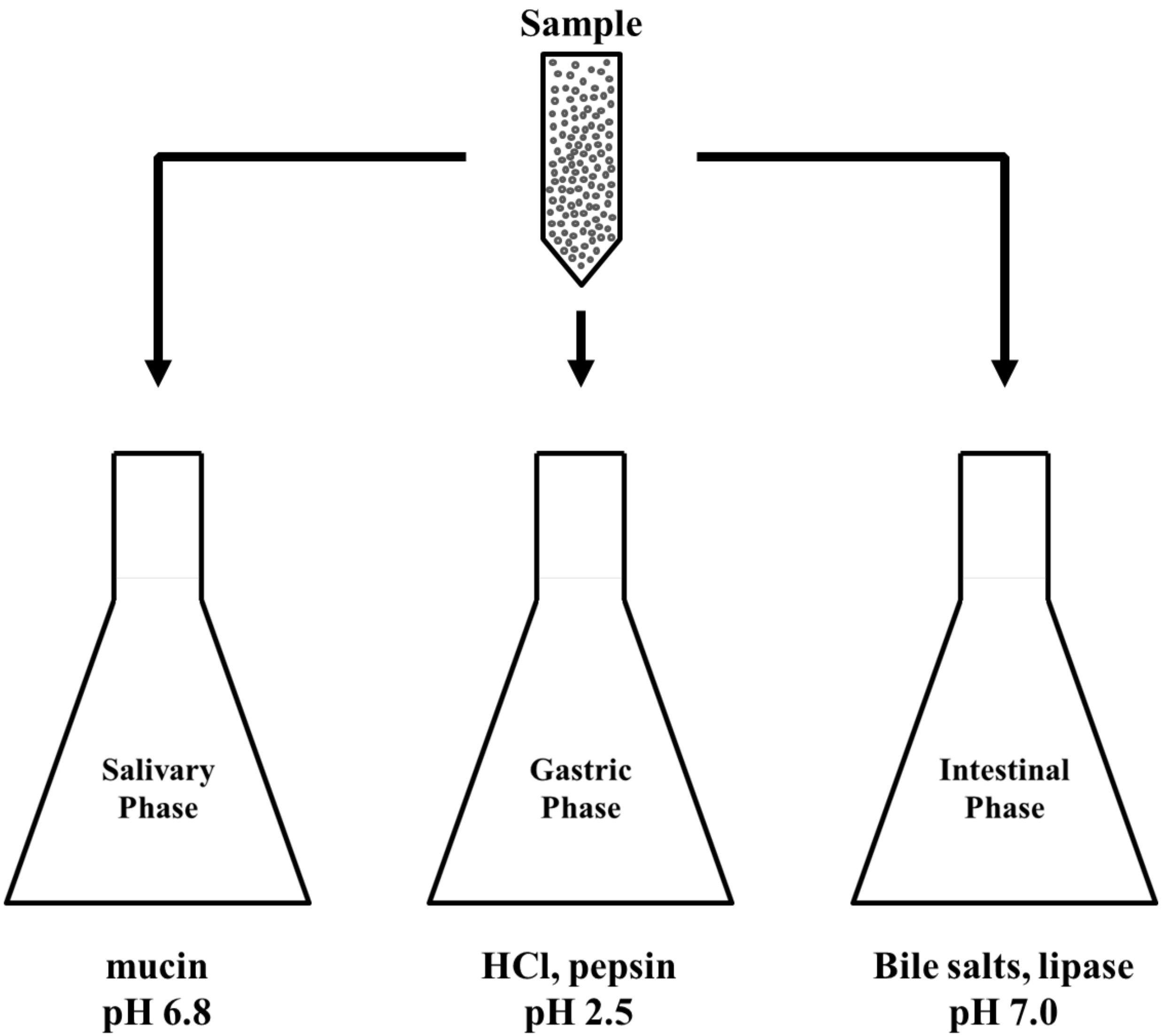

Table S1. Viability of free *Bifidobacterium longum* cells over time. Counts based on samples drop-plated on MRS agar and incubated at 37°C anaerobically. Values are shown as mean cell number ± standard error of replicate counts (n=10). Means within each column followed by the same lowercase letters are not significantly different ($p > 0.05$) from each other. Means within each row followed by the same uppercase letters are not significantly different ($p > 0.05$) from each other.

time (days)	UMA 298 free	UMA 299 free	UMA 300 free	UMA 305 free	UMA 306 free	UMA 318 free	UMA 401 free	UMA 402 free
	log CFU							
**0**	8.967 ±0.044^{aA}	9.908 ±0.043^{aAB}	10.05 ±0.018^{aBC}	10.13 ±0.084^{aBD}	9.766 ±0.060^{aACDE}	9.536 ±0.056^{aACDF}	10.28 ±0.047^{aBEF}	7.739 ±0.175^{aG}
**1**	7.770 ±0.057^{abA}	9.980 ±0.017^{aB}	9.509 ±0.072^{aB}	10.025 ±0.033^{aB}	9.592 ±0.050^{abB}	9.451 ±0.060^{aB}	3.388 ±1.130^{bC}	9.365 ±0.107^{bB}
**3**	6.683 ±0.745^{bcA}	9.667 ±0.053^{abB}	8.588 ±0.454^{aB}	7.303 ±0.813^{bA}	8.648 ±0.072^{abcBC}	7.623 ±0.027^{bAC}	1.445 ±0.736^{cD}	7.566 ±0.071^{aAC}
**5**	7.493 ±0.080^{bAB}	8.666 ±0.459^{abC}	6.672 ±0.357^{bB}	5.572 ±0.073^{cD}	8.539 ±0.074^{bcAC}	ND	4.126 ±0.101^{bF}	6.036 ±0.685^{cBD}
**7**	7.574 ±0.072^{bA}	8.579 ±0.072^{bA}	7.510 ±0.056^{bA}	3.895 ±1.298^{dB}	7.719 ±0.058^{cA}	ND	ND	5.875 ±0.113^{cD}
**10**	5.754 ±0.061^{cA}	6.549 ±0.071^{cA}	ND	ND	6.057 ±0.077^{dA}	ND	ND	8.516 ±0.065^{abC}
**14**	6.722 ±0.060^{bcA}	6.045 ±0.027^{cA}	ND	ND	6.511 ±0.120^{dA}	ND	ND	5.682 ±0.057^{cA}
**21**	3.666 ±0.624^{dA}	5.563 ±0.085^{cB}	ND	ND	4.628 ±0.523^{eAB}	ND	ND	7.270 ±0.022^{aD}
**24**	ND	3.093 ±0.517^{dB}	ND	ND	ND	ND	ND	5.833 ±0.018^{cC}
**28**	ND	ND	ND	ND	ND	ND	ND	ND
**35**	ND	ND	ND	ND	ND	ND	ND	ND

Table S2. Viability of *Bifidobacterium longum cells* encapsulated in calcium alginate over time. Counts based on samples drop-plated on MRS agar and incubated at 37°C anaerobically. Values are shown as mean cell number ± standard error of replicate counts (n=10). Means within each column followed by the same lowercase letters are not significantly different (p > 0.05) from each other. Means within each row followed by the same uppercase letters are not significantly different (p > 0.05) from each other.

time (days)	UMA 298 alg	UMA 299 alg	UMA 300 alg	UMA 305 alg	UMA 306 alg	UMA 318 alg	UMA 401 alg	UMA 402 alg
	log CFU							
**0**	8.959 ±0.049aA	10.27 ±0.054aB	9.508 ±0.029aAB	9.793 ±0.100aAB	10.07 ±0.052aBC	9.312 ±0.026aACD	10.01 ±0.069aBD	9.545 ±0.027aAB
**1**	7.058 ±0.085bA	9.694 ±0.033aB	8.018 ±0.059bCD	8.791 ±0.051bCE	9.088 ±0.087bBEF	7.406 ±0.047bAD	7.258 ±1.214bCF	8.394 ±0.059bCF
**3**	ND	9.603 ±0.020aB	7.233 ±0.075bCD	8.536 ±0.030bE	6.457 ±0.063cdC	ND	7.364 ±0.056cDF	7.202 ±0.804cdCF
**5**	ND	8.121 ±0.052bB	-	4.883 ±0.103cC	7.453 ±0.839bB	ND	3.559 ±0.782dD	7.831 ±0.021cB
**7**	ND	6.497 ±0.030cB	6.196 ±0.075cB	-	8.785 ±0.037bC	ND	-	6.355 ±0.089dB
**10**	ND	-	6.159 ±0.041cB	3.641 ±0.413dC	6.802 ±0.070cdB	ND	5.020 ±0.578eD	6.790 ±0.025dB
**14**	ND	6.298 ±0.705cB	1.571 ±0.642dC	ND	ND	ND	3.984 ±0.444dD	ND
**21**	ND	5.561 ±0.031cB	ND	ND	ND	ND	ND	ND
**24**	ND	-	ND	ND	ND	ND	ND	ND
**28**	ND	3.415 ±0.392dB	ND	ND	ND	ND	ND	ND
**35**	ND	ND	ND	ND	ND	ND	ND	ND

Table S3. Viability of *Bifidobacterium longum cells* encapsulated in chitosan-coated alginate over time. Counts based on samples drop-plated on MRS agar and incubated at 37°C anaerobically. Values are shown as mean cell number ± standard error of replicate counts (n=10). Means within each column followed by the same lowercase letters are not significantly different ($p > 0.05$) from each other. Means within each row followed by the same uppercase letters are not significantly different ($p > 0.05$) from each other.

time (days)	UMA 299 chit	UMA 300 chit	UMA 401 chit	UMA 402 chit
	log CFU			
**0**	10.31 ±0.037^{aA}	10.19 ±0.052^{aA}	10.11 ±0.042^{aA}	9.637 ±0.023^{aA}
**1**	9.764 ±0.035^{aA}	-	9.660 ±0.028^{aA}	6.424 ±1.075^{bC}
**3**	9.435 ±0.032^{abA}	8.960 ±0.061^{abA}	7.717 ±0.024^{bB}	6.638 ±0.046^{bB}
**5**	9.122 ±0.080^{abA}	8.469 ±0.046^{bAB}	7.665 ±0.028^{bB}	2.191 ±0.894^{cC}
**7**	8.356 ±0.128^{bA}	6.544 ±0.063^{cB}	2.356 ±0.788^{cC}	-
**10**	2.753 ±0.603^{cA}	-	3.194 ±0.698^{cA}	-
**14**	3.304 ±0.558^{cAB}	2.357 ±0.643^{dA}	ND	4.212 ±0.076^{dB}
**21**	ND	ND	ND	ND
**24**	ND	ND	ND	ND
**28**	ND	ND	ND	ND
**35**	ND	ND	ND	ND
